# The traditional lunch pattern is inversely correlated with body mass index in a population-based study in Brazil

**DOI:** 10.1186/s12889-017-4582-3

**Published:** 2017-07-19

**Authors:** Roberta de Oliveira Santos, Diva Aliete dos Santos Vieira, Andreia Alexandra Machado Miranda, Regina Mara Fisberg, Dirce Maria Marchioni, Valéria Troncoso Baltar

**Affiliations:** 1 Department of Nutrition, School of Public Health, University of Sao Paulo, Avenida Dr. Arnaldo, 715 – Cerqueira César. CEP, São Paulo, SP 01246-904 Brazil; 2 Department of Epidemiology and Biostatistics, Institute of Collective Health, Federal Fluminense University, Travessa Marquês de Paraná, 303 – Centro. CEP, Niterói, RJ 24030-210 Brazil

**Keywords:** Lunch, Meal, Body mass index, Obesity

## Abstract

**Background:**

The association of obesity and dietary patterns has been well documented in scientific literature; however, information on the impact of meal patterns on obesity is scarce. The objective of this study was to investigate the association of adherence to lunch patterns and body mass index (BMI) in a representative sample of individuals aged 20 years or older in Sao Paulo.

**Methods:**

Data for 933 participants were retrieved from the Health Survey of São Paulo (ISA-Capital 2008), a cross-sectional population-based survey. The usual dietary intake of individuals with at least one 24-h recall was estimated by the Multiple Source Method. The definition of lunch was self-reported by the participant. Five lunch patterns were derived from twenty-two food groups by exploratory factor analysis: Traditional, Western, Sweetened juice, Salad, and Meats. To estimate the effect of lunch patterns on BMI, we used a generalized linear model with link identity and inverse Gaussian distribution. Analyses were adjusted by age, gender, household income per capita, physical activity levels, smoking status, alcohol consumption, total energy intake, and misreporting status.

**Results:**

The greater adherence to the traditional pattern at the lunch meal was associated with lower BMI, only in insufficiently active individuals (ß = −0.78; 95% CI -1.57; −0.02).

**Conclusions:**

The traditional Brazilian lunch pattern might protect the insufficiently active individuals against obesity.

## Background

Numerous studies have examined and reported the association of environmental and behavioral factors and increased adiposity, such as intake of diets that are rich in fat and sugar and poor in fiber, vitamins and minerals, and also low levels of physical activity [1–4]. The associations between dietary patterns, which consider the complexity of diets and the potential for interactions between food components, and obesity [5, 6], have been well documented in scientific literature [7–11]. Among the findings are the association between Western pattern and being overweight or obese, and the protective role of the traditional diets [7–9]; however, studies on patterns’ impact at the level of a meal on obesity are scarce.

Considering that individuals have structured meals and combine foods within these meals, it might be important to investigate the impact of meal patterns on chronic conditions such as obesity. Dietary patterns studies capture the overall food intake of the population [7–11]; however understanding the composition of main meals (breakfast, lunch, and dinner) through its patterns clarifies details that would not be evident in a global analysis [12, 13].

Main meals are characterized as those in which the largest volume of food is usually consumed [13–15]. Among the findings of a prospective study from the United Kingdom is that the lunch meal provided the greatest proportion of the total daily energy, protein, fat and carbohydrate intake, which was consistent over time [16].

Lunch provides about 30% of daily energy value [17]; however, there is no consensus on the definition of lunch [13]. Several meal definitions have been used in the literature, and one of these definitions is the definition reported by the participant [12–14]. In this definition, the respondent identifies the name of meal as an example of lunch; the advantage of this approach is that it avoids the imposition of a complex criterion to classify different meals [13].

Due to the important daily energy contribution of lunch, it should provide adequate amounts of macro and micronutrients to assist populations in achieving dietary guidelines. In a study by Dewolfe & Millan [18], eating lunch daily was associated with higher diet quality scores that assessed compliance with the Canadian Guide to Healthy Eating. In another study that evaluated the nutritional quality of meals consumed away from home and its association with overall diet quality, the authors found that lunch consumed away from home were the worst quality when compared to lunch at home because of the higher total and saturated fat content [19]. According to Bellisle and colleagues [14], the higher peak of energy distribution and nutrient intake over an average day appeared around noontime that corresponded to lunch, the largest meal in the traditional French diet. Nonetheless, to date, we did not find any study of its association with obesity. Thus, we investigated the association between adherence to lunch patterns and body mass index (BMI).

## Methods

### Study population and design

Data were retrieved from the Health Survey of São Paulo (ISA-Capital 2008), a cross-sectional population-based survey, to assess health conditions in the city of São Paulo. This study uses complex probabilistic sampling, by conglomerates, in two stages: census tracts and household. In the first stage, the census tracts were drawn, using a probability of the number of households. In the second stage, the households were drawn, using an inverse probability of the number of households. The sample of ISA-Capital 2008 was defined for eight age domains: less than 1 year old, 1–11 years old, and three more age groups by gender: 12–19 years old (adolescents), 20–59 years old (adults), and 60 years and older (elderly adults). A minimum sample size of 300 individuals in each of the eight domains was estimated based on a prevalence of 0.5 with a standard error of 0.07 at a 5% significance level and with 1.5 design effect. A total of 3271 individuals participated in the survey and answered questions about life and socio-demographic conditions. Among these individuals, 2691 were aged 12 years or older, and they answered questions about diet; however 38% (*n* = 1029) refused to participate or changed their address/telephone and could not be reached. The sample comprised 1662 subjects (560 adolescents and 1102 adults and elderly). For the present study, 1102 who had also completed at least one 24-h recall (24HR) were included. Of those 1102 participants, 179 were not considered in the analysis because of missing information, such as lunch skipping (*n* = 32), food grouping (*n* = 17), BMI (*n* = 37), misreporting status (*n* = 1), and household per capita income (*n* = 82). The final sample was 933 individuals.

The School of Public Health of the University of São Paulo Ethics Committee approved the project. A signed written informed consent form was obtained from all participants included in this study.

### Anthropometric measurements

A structured questionnaire was used to obtain anthropometric measurements. The information of weight and height was self-reported by the participants. We calculated the BMI by dividing the weight in kilograms by the square of the height in meters. In ISA-Capital 2008, the agreement between measured and self-reported BMI was high (*r* > 0,85) [20].

### Dietary assessment

Dietary intake was assessed using two non-consecutive 24HRs collected on different weekdays, weekends, and seasons. The participation rate with two 24HRs was 50%; the first 24HR was collected via face-to-face household interviews, using the Multiple Pass Method, and the second 24HR was performed via telephone interviews, based on the computer version of the Automated Multiple Pass Method [21]. Dietary data were entered into the Nutrition Data System for Research software (version 2007, University of Minnesota, Minneapolis, MN, USA) to obtain the nutritional composition of the 24HR. The amounts of foods and beverages reported were converted into weight or volume units, and typical or specific preparations of the different Brazilian regions were provided according to national publications [22, 23].

We considered lunch the eating episode that was labeled as “lunch” by survey respondents during application of the 24HR with the following question ‘What name would you give to this meal?.’ Food items from lunch were grouped into twenty-two groups considering nutritional value, the Brazilians’ intake habits, literature data, and experience of the research team in previous studies [12, 24, 25]. The usual individual intake of each food group was estimated by the Multiple Source Method (version 1.0.1, 2011, German Institute of Human Nutrition), a statistical method developed to estimate the usual individual intake of foods, considering the intrapersonal variability [26].

Implausible dietary energy intake was estimated, using the predicted total energy expenditure method [27], and the standard deviations (SD) were calculated, using published estimations of variation in energy balance components [28], obtaining the mean value of 1 SD = 25.4%. In the present study, misreporting status was categorized as “under- (< −1 SD), plausible- (−1 SD ≤ intake ≤ + 1 SD) or over-reporting (> +1 SD).”

### Derivation of lunch patterns

The method used to derive the lunch patterns of participants has been previously described in detail [12]. Briefly, the lunch patterns were derived by exploratory factor analysis with varimax rotation, using twenty- two food groups (factor loading ≥ |0.30|). Five patterns were identified: Traditional (positive factor loadings for rice and beans; negative factor loadings for pasta); Western (positive factor loadings for soft drinks, alcoholic beverages, sweets, gnocchi/stuffed pasta, sauces/mayonnaise, and processed meats); Sweetened juice (positive factor loadings for natural juice and sugar); Salad (positive factor loadings for greens, salad dressings, and natural condiments); and Meats (positive factor loadings for eggs, poultry meat, and fish/seafood; negative for beef). These patterns explained 34.1% of the total variance of the lunch meal, with Traditional and Salad patterns explaining the greatest proportion of variance (8.4% and 7.6%, respectively).

Factor scores were estimated by multiple regression analysis, and each individual received a score for each dietary pattern. These scores indicate the degree by which each participant adheres to the pattern. Thus, to evaluate adherence, the lunch patterns were categorized according to tertiles. The group over the upper tertile represented those with the highest adherence to the pattern.

### Statistical analyses

The characteristics of the study participants were examined according to tertile groups of lunch pattern. Data are presented as the median and interquartile range (IQR) for continuous variables, and as frequencies and percentages for categorical variables.

Generalized linear models with link identity and inverse Gaussian distribution were used to examine the associations between lunch patterns and BMI. The analyses were adjusted for age (years), gender (male or female), household income per capita (US$ *per* month), physical activity levels (categorized as insufficiently or sufficiently active according to the international physical activity questionnaire validated in Brazil [29, 30]), smoking status (never smoke or former/current smoker), alcohol consumption (no or yes), usual total energy intake (kcal/day), and misreporting under-, plausible- or over-reporting). In addition, the lunch patterns were mutually adjusted in these five models.

Interaction terms of sociodemographic, economic, and lifestyle characteristics with tertile groups of lunch patterns were tested and when significant, they were maintained in models. All statistical analyses were performed, using R software version 3.2.2 and a *p* value <0.05 was considered significant.

## Results

The sample comprised 933 individuals, mean age 53.8 (SD = 18.9) years, with mean BMI 25.9 (SD = 4.7) kg/m^2^, mostly women (60.3%), non-smokers (58.2%), insufficiently active (89.7%), and approximately half of the sample did not consume alcohol (52.4%).

Sociodemographic characteristics of the study population across tertile categories of the dietary pattern scores are shown in Table 1. A greater proportion of individuals who were male, had a normal weight, were insufficiently active, with greater median total energy intake and plausible energy intake had greater adherence (T3) to the traditional pattern. A greater proportion of individuals who were female, with overweight, that were insufficiently active, with greater median total energy intake and plausible energy intake had greater adherence (T3) to the western, sweetened juice, salad and meats patterns.

**Table 1:** The main characteristics of the study participants by tertile (T) categories of dietary pattern scores in the ISA-Capital 2008

	Traditional			Western			Sweetened juice			Salad			Meats		
	T1	T2	T3	T1	T2	T3	T1	T2	T3	T1	T2	T3	T1	T2	T3
Sex, n (%)															
Male	87 (28.34)	94 (29.84)	189 (60.77)	100 (32.47)	120 (39.09)	150 (47.17)	120 (38.96)	129 (41.88)	121 (38.17)	137 (45.21)	111 (35.35)	122 (38.61)	146 (47.10)	110 (35.14)	114 (36.77)
Female	220 (71.66)	221 (70.16)	122 (39.23)	208 (67.53)	187 (60.91)	168 (52.83)	188 (61.04)	179 (58.12)	196 (61.83)	166 (54.79)	203 (64.65)	194 (61.39)	164 (52.90)	203 (64.86)	196 (63.23)
Body mass index (kg/m^2^), median (IQR)	26.04 (23.31, 29.07)	25.39 (22.83, 28.91)	24.93 (22.19, 27.99)	25.71 (22.88, 29.07)	25.39 (22.83, 28.65)	25.08 (22.53, 28.13)	25.53 (22.66, 28.55)	25.62 (23.18, 29.14)	25.25 (22.41, 28.40)	24.73 (22.15, 28.41)	26.00 (23.31, 29.07)	25.52 (23.09, 28.40)	25.39 (22.40, 28.76)	25.59 (22.94, 28.91)	25.32 (22.83, 28.13)
Physical activity levels, n (%)															
Insufficiently active	269 (87.62)	286 (90.79)	282 (90.68)	282 (91.56)	275 (89.58)	280 (88.05)	269 (87.34)	273 (88.64)	295 (93.06)	275 (90.76)	289 (92.04)	273 (86.39)	290 (93.55)	273 (87.22)	274 (88.39)
Sufficiently active	38 (12.38)	29 (9.21)	29 (9.32)	26 (8.44)	32 (10.42)	38 (11.95)	39 (12.66)	35 (11.36)	22 (6.94)	28 (9.24)	25 (7.96)	43 (13.61)	20 (6.45)	40 (12.78)	36 (11.61)
Total energy intake (kcal/day), median (IQR)	1352 (1029, 1868)	1409 (1072, 1849)	1900 (1413, 2439)	1368 (1079, 1815)	1487 (1099, 1998)	1813 (1303, 2469)	1459 (1097, 2034)	1494 (1083, 2014)	1653 (1229, 2245)	1501 (1106, 2065)	1489 (1091, 2007)	1598 (1237, 2137)	1572 (1207, 2150)	1484 (1086, 1963)	1582 (1110, 2178)
Misreporting status, n (%)															
Under-reporting	99 (32.25)	74 (23.49)	40 (12.86)	88 (28.57)	83 (27.04)	42 (13.21)	74 (24.03)	75 (24.35)	64 (20.19)	73 (24.09)	77 (24.52)	63 (19.94)	57 (18.39)	82 (26.20)	74 (23.87)
Plausible-reporting	169 (55.05)	202 (64.13)	202 (64.95)	188 (61.04)	183 (59.61)	202 (63.52)	199 (64.61)	189 (61.36)	185 (58.36)	175 (57.76)	197 (62.74)	201 (63.61)	200 (64.52)	186 (59.42)	187 (60.32)
Over-reporting	39 (12.70)	39 (12.38)	69 (22.19)	32 (10.39)	41 (13.36)	74 (23.27)	35 (11.36)	44 (14.29)	68 (21.45)	55 (18.15)	40 (12.74)	52 (16.46)	53 (17.10)	45 (14.38)	49 (15.81)

Sao Paulo. Brazil. (*n* = 933)

The insufficiently active individuals presented BMI mean of 25.9 (SD = 4.8) kg/m^2^; they were mostly women (62.3%) with total energy intake mean of 1688.5 (SD = 769.8) kcal. Sufficiently active individuals were predominately male (56.3%), with BMI mean of 25.9 (SD = 4.1) kg/m^2^ and total energy intake mean of 1819.8 (SD = 970.6) kcal (data not shown).

Generalized linear regression models between BMI and the tertile categories of the dietary pattern scores are shown in Table 2. In the crude model, traditional (T3) and salad patterns (T2) were inversely and directly associated with BMI, respectively. On the other hand, in the adjusted analysis, a greater adherence to the traditional pattern was associated with lower BMI, only for insufficiently active individuals. A significant interaction between the dietary patterns and physical activity levels was detected only for the traditional pattern. Therefore, in insufficiently active individuals the third tertile of traditional pattern was associated with a 0.78 lower BMI in average, when compared with the first tertile (reference tertile). No association was observed when evaluating sufficiently active individuals. There was no association between the other patterns and BMI after adjustment.

**Table 2:** Regression coefficients for the association of the dietary patterns with body mass index in the study population

Lunch Patterns				
*Crude model*			β	95% CI
Traditional		Pattern (ref. 1st tertile)		
		2nd tertile	−0.27	−1.01; 0.48
		3rd tertile	−1.07	−1.80;-0.34
Western		Pattern (ref. 1st tertile)		
		2nd tertile	0.01	−0.74; 0.75
		3rd tertile	−0.38	−1.11; 0.35
Sweetened juice		Pattern (ref. 1st tertile)		
		2nd tertile	0.22	−0.53; 0.97
		3rd tertile	−0.56	−1.29; 0.17
Salads		Pattern (ref. 1st tertile)		
		2nd tertile	0.78	0.04; 1.52
		3rd tertile	0.46	−0.27; 1.19
Meats		Pattern (ref. 1st tertile)		
		2nd tertile	0.22	−0.52; 0.96
		3rd tertile	−0.16	−0.90; 0.56
*Model adjusted* ^a^			β	95% CI
Traditional	Insufficiently active	Pattern (ref. 1st tertile)		
		2nd tertile	−0.50	−1.23, 0.24
		3rd tertile	−0.78	−1.57, −0.02
	Sufficiently active	2nd tertile	1.99	−0.99, 5.05
		3rd tertile	0.84	−2.09, 3.82
Western		Pattern (ref. 1st tertile)		
		2nd tertile	−0.07	−0.77, 0.63
		3rd tertile	0.14	−0.58, 0.85
Sweetened juice		Pattern (ref. 1st tertile)		
		2nd tertile	0.17	−0.54, 0.87
		3rd tertile	−0.29	−0.99, 0,40
Salads		Pattern (ref. 1st tertile)		
		2nd tertile	0.37	−0.33, 1.07
		3rd tertile	0.06	−0.62, 0.75
Meats		Pattern (ref. 1st tertile)		
		2nd tertile	0.01	−0.68, 0.70
		3rd tertile	−0.29	−0.98, 0.39

^a^Adjusted for age, sex, household per capita income, physical activity levels, smoking status, alcohol consumption, total energy intake, misreporting status and lunch patterns

ISA-Capital 2008. Sao Paulo. Brazil

## Discussion

Our findings from this cross-sectional study suggest that the traditional pattern based on rice and beans in the lunch meal was inversely associated with BMI in the insufficiently active individuals in the generalized linear regression models analysis. We observed no association between the other dietary patterns and BMI.

The traditional pattern composed of Brazilian staple foods, such as rice and beans, was also reported in previous studies that were conducted in the Brazilian regions [7, 31–34]. Marchioni et al. [31], using data from the cross-sectional national Household Budget Survey (HBS), described that the overall pattern denominated traditional had significant contributions from food groups used for domestic food preparation and cooking of Brazilian traditional dishes, such as rice, beans, cassava, flour, milk and sugar; and these are the similar foods to the pattern found in our study. This combination is considered a healthy dish; and the intake of rice and beans is promoted by the Brazilian Health Ministry [35].

According to Cunha et al. [8], in a population-based cross-sectional study conducted in Rio de Janeiro State, the traditional pattern characterized by the consumption of rice and beans had a protective role against weight gain and a beneficial effect on body mass index and waist circumference among women. The study by Castro et al. [25] revealed that high adherence to the traditional pattern was associated with a reduction in body weight and waist circumference, by mediation of serum leptin, which suggests a protective role of this pattern against weight gain and abdominal fat accumulation. Other studies in Brazil and elsewhere also observed an inverse association of this pattern with BMI [7, 10, 11, 36]. Possible explanations for this association are the low glycemic index (GI), low-energy density, low-fat intake, and the high-fiber intake due to the consumption of beans [7, 8]. A study by Sugiyama et al. [37] that tested beans showed low GI values and a GI lowering effect when beans were consumed with a carbohydrate meal. Thompson et al. [38] demonstrated that beans in *P. vulgaris* species, broadly consumed by many countries, including those in Latin America, United States of America, and countries within the Mediterranean and Middle East, attenuate the glycemic response to rice, a commonly consumed high GI food.

In addition, beans are the main source of total fiber intake in São Paulo [39], and the combination of rice and beans accounted for the majority of total fiber available in Brazilian households [40]. The protective effect of dietary fiber on excess body weight has been shown in systematic reviews [41, 42], cross-sectional studies [25, 43, 44], and in a prospective cohort study [45]. Moreover, the consumption of beans can also be a marker of a more consistent home-prepared diet, which has been shown to be associated with a reduced intake of fats and high-energy density foods [7]. Furthermore, rice and beans are also low-energy density foods that contribute to the bulk of the Brazilian diet [7].

Despite the fact that the dietary intake has a direct relationship with the BMI, other modifiable risk factor, such as physical activity should be taken into account, since weight gain in adults and elderly adults depends on the balance between the energy expenditure and dietary intake [46]. The inclusion of interaction term between leisure-time physical activity and tertile groups of lunch pattern in our models allowed the detection of the inverse association between traditional pattern and BMI. The greater adherence to the traditional pattern seems to be related to a beneficial effect on the BMI, especially in insufficiently active individuals. We observed that these individuals presented less energy intake; however, it should be highlighted that the analyses were controlled for sex, total energy intake, alcohol consumption, per capita income, and misreporting.

Some points should be considered in interpreting the findings of the current study. Firstly, our data are related to the cross sectional study design that does not allow definitive establishment of causal inference and can only provide assumptions about the association between dietary patterns and BMI. Thus, our findings need to be confirmed in a future prospective study. In regards to the diet intake, the 24HR responses depend on the respondents’ memory and do not measure habitual diet. However, this instrument was repeated in a sub-sample of participants, allowing us to estimate the usual intake, adjusting for intra-individual variance [12]. Despite adjusting for potential confounders, residual confounding might be present. Several subjective and arbitrary decisions in the use of factor analysis need to be considered. Exploratory factor analysis is a method involving decision-making by researchers at various stages of the modeling process, such as during food grouping, choosing the factor rotation method, defining criteria for retention of factors, and establishing a cut-off point for factor loadings [12]. However, in an effort to ensure methodological rigor, the authors adopted analytical procedures widely accepted in Nutritional Epidemiology for the study of dietary patterns [47].

Nevertheless, the principal strength of this study is that it is the first study examining the associations between lunch meal dietary patterns and BMI. Moreover, we have adjusted for potential confounders and checked several possible interaction effects.

## Conclusion

The greater adherence to the traditional pattern in the lunch meal was associated with lower BMI only in insufficiently active individuals. This relationship was not evident in active individuals. In this context, the consumption of traditional Brazilian foods might have potentially beneficial effects against being overweight among adults and the elderly adults that already have the risk factor of not being active. The promotion of traditional pattern at lunch could then contribute to control obesity, especially for insufficiently active people.

## Abbreviations

24HR: 24-h recall
BMI: Body Mass Index
CI: Confidence Interval
GI: Glycemic Index
IQR: Interquartile Range
SD: Standard Deviation
T: Tertile
